# A lightweight triple-modal fusion network for progressive mild cognitive impairment prediction in Alzheimer's disease

**DOI:** 10.3389/fnins.2025.1637291

**Published:** 2025-08-26

**Authors:** Xiangyu Shen, Xiangyang Hu, Renfeng Zhang, Yunzhan Fu, Jiamin Xu, Degang Lyu, Hongbiao Xie, Deen Shi, Changsheng Shi, Lisi Li, Yuantong Gao

**Affiliations:** ^1^Hangzhou Dianzi University, Hangzhou, China; ^2^Department of Laboratory Medicine, Shandong Provincial Hospital Affiliated to Shandong First Medical University, Jinan, China; ^3^Department of Radiation Oncology, Shenzhen People's Hospital (The Second Clinical Medical College, Ji'nan University, The First Affiliated Hospital of Southern University of Science and Technology), Shenzhen, China; ^4^School of Information Technology, Zhejiang Institute of Economics and Trade, Hangzhou, China; ^5^Department of Radiology, The Third Affiliated Hospital of Wenzhou Medical University, Wenzhou, China; ^6^Department of Interventional Vascular Surgery, The Third Affiliated Hospital of Wenzhou Medical University, Wenzhou, China; ^7^Shenzhen Hospital of Southern Medical University, Shenzhen, China

**Keywords:** Alzheimer's disease, mild cognitive impairment, triple-modal fusion, lightweight neural network, attention mechanism, integrated gradients

## Abstract

**Introduction:**

As a progressive neurodegeneration, Alzheimer's disease (AD) represents the primary etiology of dementia among the elderly. Early identification of individuals with mild cognitive impairment (MCI) who are likely to convert to AD is essential for timely diagnosis and therapeutic intervention. Although multimodal neuroimaging and clinical data provide complementary information, existing fusion models often face challenges such as high computational complexity and limited interpretability.

**Methods:**

To address these limitations, we introduce TriLightNet, an innovative lightweight triple-modal fusion network designed to integrate structural MRI, functional PET, and clinical tabular data for predicting MCI-to-AD conversion. TriLightNet incorporates a hybrid backbone that combines Kolmogorov-Arnold Networks with PoolFormer for efficient feature extraction. Additionally, it introduces a Hybrid Block Attention Module to capture subtle interactions between image and clinical features and employs a MultiModal Cascaded Attention mechanism to enable progressive and efficient fusion across the modalities. These components work together to streamline multimodal data integration while preserving meaningful insights.

**Results:**

Extensive experiments conducted on the Alzheimer's Disease Neuroimaging Initiative (ADNI) dataset demonstrate the effectiveness of TriLightNet, showcasing superior performance compared to state-of-the-art methods. Specifically, the model achieves an accuracy of 81.25%, an AUROC of 0.8146, and an F1-score of 69.39%, all while maintaining reduced computational costs.

**Discussion:**

Furthermore, its interpretability was validated using the Integrated Gradients method, which revealed clinically relevant brain regions contributing to the predictions, enhancing its potential for meaningful clinical application. Our code is available at https://github.com/sunyzhi55/TriLightNet.

## 1 Introduction

Alzheimer's disease (AD) is a progressive neurodegenerative disorder characterized by irreversible cognitive decline, significantly impacting the daily functioning of affected individuals (Wen et al., 2020; Francesconi et al., 2025). The Alzheimer's Association reports that over 50 million people globally are affected by AD, with projections indicating that the number of patients in the United States will more than double by 2050 (Moradi et al., 2015). As AD advances, individuals often suffer from severe deterioration in cognitive functions, including memory loss, language difficulties, and impaired reasoning (Grigas et al., 2024). The prodromal stages of AD, categorized by the severity of cognitive decline, include subjective cognitive decline (SCD) and mild cognitive impairment (MCI) (Elazab et al., 2024). Patients in the MCI stage already show noticeable cognitive deficits. Currently, there is no effective treatment to reverse AD or MCI, and only a limited number of medications can alleviate symptoms. In clinical practice, progressive mild cognitive impairment (pMCI) refers to patients who are likely to convert to AD within ~3 years, whereas stable MCI (sMCI) describes those whose cognitive condition remains unchanged during the same period (Gaser et al., 2013). Accurately predicting pMCI is crucial for early intervention, which can delay the onset of AD.

Advances in neuroimaging have significantly enhanced our ability to gather detailed anatomical and functional information about the brain using techniques such as magnetic resonance imaging (MRI) and positron emission tomography (PET). MRI offers high-resolution structural details, distinguishing between gray and white matter, while PET detects functional changes in brain metabolism, providing insights into neurodegenerative processes. Leveraging this data, computer-aided diagnostic (CAD) systems have been developed to aid in the early differentiation between pMCI and sMCI (El-Gamal et al., 2021). Alongside imaging data, clinical information, including demographic details, laboratory test results, and neurological assessments, offers valuable insights into AD. Building on this wealth of information, machine learning (ML) techniques have been widely applied to analyze clinical data related to AD. For instance, Moradi et al. (2015) utilized a random forest (RF) classifier to predict the early conversion from MCI to AD. Similarly, Mathew et al. (2016) integrated principal component analysis, discrete wavelet transform, and support vector machines for AD classification. While these traditional ML approaches have shown promise, they heavily depend on handcrafted features, which require considerable domain expertise and are often influenced by subjective interpretations.

Recently, deep learning techniques such as ResNet (He et al., 2016), EfficientNet (Tan and Le, 2019), and Vision Transformer (ViT) (Dosovitskiy et al., 2020) have been increasingly applied to AD diagnostic tasks. Wang et al. (2024) introduced the HOPE framework, which leverages MRI features from various disease stages to predict the conversion from MCI to AD with promising results. (Zhang et al. 2025a) developed a spatiotemporal transformer-based approach for constructing asynchronous functional brain networks. Several studies have shown significant progress in MRI-based AD diagnosis (Atitallah et al., 2024; Lei et al., 2024; Jabason et al., 2025; Haq et al., 2025). However, relying solely on MRI does not capture crucial metabolic information in the brain, underscoring the limitations of single-modality approaches. To address these shortcomings, Kang et al. (2023) introduced the Visual Attribute Prompt Learning (VAPL) to integrate MRI with clinical tabular data, while Duenias et al. (2025) proposed the hyperfusion framework, which combines medical imaging with clinical features. The fusion of MRI and PET has also emerged as a common multimodal strategy, providing a comprehensive understanding of brain pathology through structural and functional imaging. Li et al. (2025) presented the Diamond framework based on ViT, using dual attention mechanisms to model inter-modal similarities. Other methods, such as MMGPL (Peng et al., 2024) and MDL (Qiu et al., 2024), have also achieved competitive performance. Moreover, the problem of missing modalities is commonly encountered in real-world scenarios, and previous studies have also explored this issue (Liu et al., 2022; Hu et al., 2025). Our work is currently conducted under the setting of complete modalities, and addressing modality missing will be a key direction for future research.

Many current models face challenges in effectively integrating more than two modalities, complicating comprehensive diagnoses that require structural imaging, functional imaging, and clinical data. To tackle this issue, Li et al. (2023) introduced the IMF framework, which enhances inter-modal interactions through a two-stage fusion design. The Modality-Flexible Framework offers adaptive diagnosis using diverse clinical data (Zhang et al., 2025b), while the longitudinal prediction method incorporates modality uncertainty to boost robustness (Dao et al., 2025). Innovative approaches have also emerged, such as the adversarial learning (Baytaş, 2024) and the flexible Mixture-of-Experts architecture (Yun et al., 2024). Despite these advancements, current multimodal fusion methods often rely heavily on deep convolutional layers and repetitive attention mechanisms, resulting in high computational overhead and increased model complexity. Streamlining these processes remains a critical area of research to enhance efficiency without sacrificing diagnostic accuracy.

To overcome the limitations of current models, we propose TriLightNet, a novel and lightweight triple-modal framework designed for predicting the conversion from MCI to AD. This model effectively integrates structural MRI (sMRI), Fluorodeoxyglucose PET (FDG-PET), and clinical tabular data while maintaining low computational demands. The main contributions of this work are summarized as follows:

- We develop a new backbone network, blending the representational power of Kolmogorov-Arnold Networks (KAN) (Liu et al., 2024) with the efficiency of PoolFormer (Yu et al., 2022). This fusion supports compact and robust feature extraction from clinical data, crucial for medical applications where computational resources may be limited.
- We present the Hybrid Block Attention Module (HBAM), designed for AD diagnostic tasks, capturing intricate interactions between imaging modalities and clinical tabular variables. This module allows the model to take into account both spatial brain patterns and essential clinical indicators such as cognitive scores and patient history.
- We propose the MultiModal Cascaded Attention (MMCA), a scalable and memory-efficient fusion strategy inspired by Cascaded Group Attention (CGA) (Liu et al., 2023). This mechanism progressively aggregates multimodal features, enhancing cross-modal synergy and addressing challenges related to modality imbalance, often encountered in real-world AD datasets.
- We perform comprehensive experiments using benchmark AD datasets, including comparative experiments, ablation studies, and interpretability analyses. The results show that TriLightNet not only surpasses existing multimodal baselines in accuracy and efficiency but also offers clinically meaningful visualizations that can facilitate medical decision-making.

## 2 Related work

### 2.1 Neural network backbones

Traditional image encoders, such as convolutional neural networks (CNNs) and Vision Transformer (ViT) (Dosovitskiy et al., 2020), have achieved remarkable performance across a wide range of visual tasks. While CNNs like ResNet are known for their efficiency and effectiveness, ViT typically needs higher computational resources and larger model sizes (He et al., 2016). To address these limitations, Yu et al. (2022) proposed PoolFormer, which eschews attention mechanisms in favor of spatial pooling and global token mixing, achieving competitive performance with reduced computational demands.

Recently, Liu et al. (2024) introduced the KAN, a novel architecture based on the Kolmogorov-Arnold representation theorem. KAN replaces the linear transformations in traditional multilayer perceptron (MLP) with learnable spline-based functions, offering a flexible and interpretable modeling framework with strong approximation capabilities, particularly beneficial in low-data scenarios and for capturing complex nonlinear relationships. Several studies have explored KAN in Alzheimer's disease. For example, Verma et al. (2025) combined KAN with Visual Geometry Group (VGG) for Alzheimer's disease prediction and achieved promising results. Others have integrated KAN with graph neural networks to further enhance modeling capabilities (Wang, 2025; Ding et al., 2025). In addition to its role as a classifier, KAN has recently been applied to tabular feature extraction tasks and demonstrated notable effectiveness (Poeta et al., 2024; Eslamian et al., 2025; Gao et al., 2024).

Building on these advancements, we propose a hybrid neural backbone that combines the architectural efficiency of PoolFormer with the functional expressiveness and interpretability of KAN. Specifically, our design utilizes PoolFormer's spatial feature aggregation capabilities and enhances representation learning with KAN-based modules, providing a robust and efficient framework for processing clinical tabular data.

### 2.2 Attention mechanism development

In visual tasks, traditional attention mechanisms include Squeeze and Excitation Networks (SENet) (Hu et al., 2018) and Convolutional Block Attention Modules (CBAM) (Woo et al., 2018). In particular, CBAM applies channel-wise attention and spatial attention sequentially to enhance feature representations by highlighting useful features and diminishing less important ones. More recently, Liu et al. (2023) proposed the CGA mechanism, a memory-efficient attention scheme that divides input tokens into separate groups and performs cascaded cross-group attention, greatly reducing computational complexity while preserving expressive power .

For AD analysis, attention mechanisms have also been explored. For instance, Li et al. (2025) combined self-attention and bi-attention to effectively fuse features from MRI and PET modalities. Similarly, Kang et al. (2023) employed cross-attention to integrate MRI data with clinical features. However, most existing approaches do not adequately consider the unique structural characteristics of each modality, particularly the disparity between high-dimensional imaging data and low-dimensional tabular data. Moreover, the high computational cost of attention is also a problem.

To address these limitations, we introduce two innovative attention modules designed for multimodal AD analysis. The first is HBAM, which extends the CBAM architecture to facilitate bidirectional attention between image and tabular modalities. The second is the MMCA, which adapts the CGA mechanism to support efficient and effective cross-modal interaction. Together, these modules form the backbone of our proposed framework, enabling precise and computationally efficient fusion of heterogeneous medical data.

### 2.3 Model interpretability techniques

Interpretability is essential in medical image analysis. Among various explainability methods (Kohlbrenner et al., 2020; Selvaraju et al., 2017; Sundararajan et al., 2017), Integrated Gradients (IG) (Sundararajan et al., 2017) has been widely adopted due to its solid theoretical foundations and practical effectiveness, which stands out as a path-based attribution method that overcomes the limitations of standard gradient-based explanations, such as saturation and noise. In the application of neuroscience in dementia detection, previous studies have successfully employed the IG attribution method (Gryshchuk et al., 2025; Wang et al., 2023). Following this line of research, we adopt IG in our study to investigate feature contributions across multimodal inputs.

For a given model *F*, an input ***x***, and a baseline input ***x*****′** (often a zero vector), IG calculates the attribution for each input dimension as the path integral of the gradients along a straight-line path from the baseline to the actual input:


(1) $$\begin{array}{l} {IG}_{i}\left(x\right)=\left(x_{i}-x_{i}^{\prime }\right)\times \int_{\alpha =0}^{1}\frac{\partial F\left(x_{i}^{\prime }+\alpha \times \left(x_{i}-x_{i}^{\prime }\right)\right)}{\partial x_{i}^{\prime }}d\alpha , \end{array}$$

where ***IG***_*i*_(***x***) represents the attribution of the *i*-th feature. Essentially, IG quantifies each input feature's contribution to the change in the model output from the baseline to the actual input. This approach is particularly suitable for high-dimensional medical data, providing pixel-level attributions for image modalities and feature-level attributions for clinical inputs.

## 3 Materials and methods

### 3.1 Materials

This study utilizes data from the Alzheimer's disease Neuroimaging Initiative (ADNI), drawing specifically from the ADNI-1 and ADNI-2 datasets (Mueller et al., 2005). The ADNI cohort includes individuals diagnosed with AD, MCI, and Normal Controls (NC). To avoid duplication, subjects appearing in both datasets were excluded from ADNI-2. We used T1-weighted sMRI, FDG-PET imaging, and clinical data, categorizing subjects into pMCI and sMCI groups. Demographic information is detailed in Table 1.

**Table 1:** Characteristics of the datasets used in experiments.

**Variable**	**ADNI1 (Mueller et al.**, **2005****)**				**ADNI2 (Mueller et al.**, **2005****)**			
	**AD**	**pMCI**	**sMCI**	**NC**	**AD**	**pMCI**	**sMCI**	**NC**
Number (M/F)	88/83	90/61	136/72	103/104	89/67	43/38	156/125	132/165
Age	75.35 ± 7.47	74.63 ± 7.18	74.75 ± 7.63	75.92 ± 5.12	74.75 ± 8.09	72.60 ± 7.27	71.29 ± 7.43	72.80 ± 6.01
Education	14.64 ± 3.19	15.66 ± 2.92	15.61 ± 3.11	15.91 ± 2.87	15.72 ± 2.75	16.29 ± 2.55	16.31 ± 2.61	16.61 ± 2.5
CDR-SB	4.32 ± 1.58	1.85 ± 0.98	1.38 ± 0.75	0.03 ± 0.12	4.51 ± 1.67	2.18 ± 0.95	1.33 ± 0.82	0.04 ± 0.15
MMSE	23.23 ± 2.03	26.59 ± 1.7	27.33 ± 1.77	29.14 ± 0.98	23.12 ± 2.07	27.1 ± 1.82	28.21 ± 1.63	28.99 ± 1.26

Given the challenges of missing PET data and class imbalance between pMCI and sMCI in both datasets, we focused on subjects with complete multimodal data, comprising MRI, PET, and clinical features. Therefore, we merge the ADNI-1 and ADNI-2 cohorts into a single dataset. The finalized dataset comprises 512 subjects: 149 pMCI and 363 sMCI. The dataset was randomly split into training and testing sets with a 4:1 ratio. The training set included 119 pMCI and 290 sMCI. The testing set comprised 30 pMCI and 73 sMCI.

All MRI images underwent preprocessing, including intensity normalization, skull stripping, and normalization to Montreal Neurological Institute (MNI) space. FDG-PET images were similarly processed through intensity normalization, normalization to MNI space, and co-registration with MRI images. All images were resized to a resolution of 96 × 128 × 96. For clinical data, similar to previous studies (Hu et al., 2025; Duenias et al., 2025; Kang et al., 2023) and based on clinical experience from doctors, we selected seven features. Among them, age, gender, and education belong to demographic attributes, while ApoE4 status, phosphorylated tau 181 (P-tau 181) and total tau (T-tau) are categorized as cerebrospinal fluid (CSF) biomarkers. The last attribute is a composite measure derived from 18F-fluorodeoxyglucose (FDG) and florbetapir (AV45) PET scans. It is worth noting that cognitive scores were excluded because they are directly related to the diagnosis of Alzheimer's disease and thus were excluded to avoid introducing bias into the prediction of disease progression.

### 3.2 Methodology

This section details the architecture of TriLightNet, our lightweight triple-modal fusion network for predicting MCI-to-AD progression. We first describe the feature extraction from MRI, PET, and clinical data. For imaging modalities, ResNet is employed to capture spatial features, and the tabular encoder combines KAN with PoolFormer to enable efficient and expressive representation learning from clinical variables. Subsequently, we introduce the HBAM for nuanced image-clinical feature interaction, followed by the MMCA for efficient progressive fusion of all modalities. Finally, we discuss the loss function used for model training.

#### 3.2.1 Feature extraction

In our study, we utilize ResNet as the encoder for medical imaging data such as MRI and PET scans to extract deep spatial features effectively. For clinical tabular data, we developed a specialized encoder that combines the KAN module with the PoolFormer architecture. The KAN module provides a flexible structure and high computational efficiency, and PoolFormer replaces the self-attention mechanisms typically found in traditional Transformer with simple pooling operations.

A general KAN network consists of *L* layers. Given an input $x_{0}\in {\mathbb{R}}^{n_{0}}$, its output is defined as:


(2) $$\begin{array}{l} \text{KAN}\left(x_{0}\right)=\left(\Phi_{L-1}○\cdots ○\Phi_{1}○\Phi_{0}\right)\left(x_{0}\right), \end{array}$$

where each Φ_*l*_ denotes a nonlinear transformation. Typically, B-spline curves are used as the nonlinear activation functions due to their ability to precisely approximate low-dimensional functions, thereby enhancing network accuracy. A B-spline function is defined as a piecewise polynomial expressed as a linear combination of basis functions:


(3) $$\begin{array}{l} S_{n,t}\left(x\right)=\overset{n-1}{\underset{i=0}{\sum }}c_{i}B_{i}\left(x\right), \end{array}$$

where *n* is the number of control points, *t* is the knot vector, ***c***_*i*_ are the control point coefficients, and ***B***_*i*_(*x*) are the basis functions.

Let the original clinical input be denoted as $I_{cli}\in {\mathbb{R}}^{C_{ori}}$. In our implementation, the feature extraction process for clinical data involves two main stages. First, we use a single layer KAN module with SiLU-based spline activations to map the input ***I***_*cli*_ from its original dimension to a higher-dimensional latent feature space. This step generates an initial feature representation, which we denote as ***H***_0_:


(4) $$\begin{array}{l} H_{0}=\text{KAN}\left(I_{cli}\right). \end{array}$$

Subsequently, this initial representation ***H***_0_ is fed into a sequence of *M* stacked PoolFormer blocks to further refine the features. Each PoolFormerBlock consists of an average pooling operation and an MLP, both integrated with residual connections and layer normalization. The transformation within the *m*-th block (for *m* = 1, …, *M*) is defined as:


(5) $$\begin{array}{l} H_{m}^{\prime }=H_{m-1}+\text{AvgPool}\left(\text{LayerNorm}\left(H_{m-1}\right)\right), \end{array}$$

(6) $$\begin{array}{l} H_{m}=H_{m}^{\prime }+\text{MLP}\left(\text{LayerNorm}\left(H_{m}^{\prime }\right)\right), \end{array}$$

where ***H***_*m*−1_ is the input to the *m*-th block and ***H***_*m*_ is its output.

#### 3.2.2 Hybrid Block Attention Module

**Figure 1:**
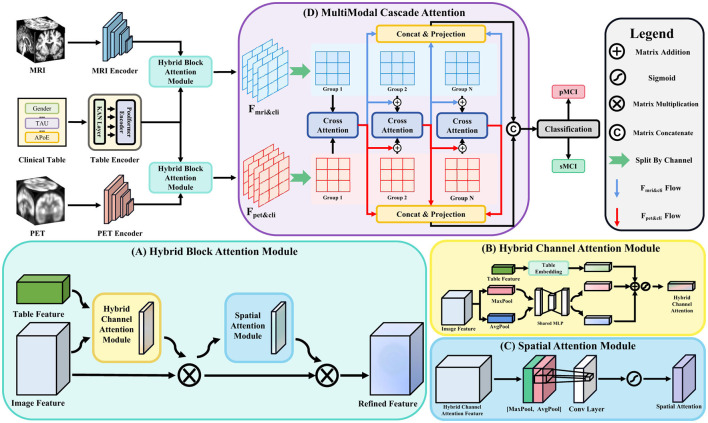
The overall architecture of TriLightNet, including **(A)** Hybrid Block Attention Module and **(D)** MultiModal Cascaded Attention. Among these, Hybrid Block Attention Module is composed of two sub-modules: **(B)** Hybrid Channel Attention Module, responsible for adaptively assigning weights to channel-level interactions between table and image features; and **(C)** Spatial Attention Module, which captures and enhances local spatial dependencies.

We introduce HBAM, which builds upon the CBAM by extending its attention mechanism to effectively integrate multimodal inputs, specifically image and clinical features. As depicted in Figure 1A, HBAM processes both image and tabular features. Let $F_{img}\in {\mathbb{R}}^{C_{img}\times D\times H\times W}$ represent the feature tensor extracted from an image encoder, and $F_{cli}\in {\mathbb{R}}^{C_{cli}}$ denotes the vectorized representation of clinical tabular data. The objective is to refine ***F***_*img*_ by leveraging complementary information from ***F***_*cli*_, thereby enhancing the overall feature representation.

##### 3.2.2.1 Hybrid Channel Attention Module

The Hybrid Channel Attention Module (HCAM) is an essential part of HBAM, specifically crafted to merge information from both image and clinical features at the channel level. As depicted in Figure 1B, HCAM refines feature representation by utilizing complementary data from these two modalities.

Initially, the image feature tensor ***F***_*img*_ and the clinical feature vector ***F***_*cli*_ are processed. The clinical feature is embedded into a format compatible with the image feature using a linear embedding layer:


(7) $$\begin{array}{l} E_{cli}=\text{Embed}\left(F_{cli}\right), \end{array}$$

where Embed(·) is a linear layer that projects ***F***_*cli*_ from ${\mathbb{R}}^{C_{cli}}$ to ${\mathbb{R}}^{C_{img}}$.

Subsequently, Global Average Pooling (GAP) and Global Max Pooling (GMP) are applied to the image feature tensor to yield two vectors that capture different aspects of the image feature:


(8) $$\begin{array}{l} F_{avg}=\text{GAP}\left(F_{img}\right)\in {\mathbb{R}}^{C_{img}},\;F_{max}=\text{GMP}\left(F_{img}\right)\in {\mathbb{R}}^{C_{img}}. \end{array}$$

These pooled vectors are passed through a shared MLP to generate channel attention weights:


(9) $$\begin{array}{l} M_{avg}=\text{MLP}\left(F_{avg}\right),\;M_{max}=\text{MLP}\left(F_{max}\right). \end{array}$$

The ***M***_*avg*_, ***M***_*max*_, and ***E***_*cli*_ are combined using element-wise addition and normalization to produce the hybrid channel attention map ***M***_*hc*_:


(10) $$\begin{array}{l} M_{hc}=\sigma \left(M_{avg}+M_{max}+E_{cli}\right), \end{array}$$

where σ is the sigmoid function.

Finally, the hybrid channel attention map ***M***_*hc*_ is applied to the original image feature tensor ***F***_*img*_ through channel-wise multiplication:


(11) $$\begin{array}{l} F_{hc}=M_{hc}⊗F_{img}, \end{array}$$

where ⊗ indicates channel-wise multiplication.

This design allows HCAM to effectively blend information from both image and tabular features, enhancing the feature representation by highlighting informative channels and suppressing less useful ones. The refined feature tensor ***F***_*hc*_ is then forwarded to the Spatial Attention Module for further enhancement.

##### 3.2.2.2 Spatial attention module

Following the refinement at the channel level, we employ a spatial attention module akin to that in CBAM to further emphasize important spatial regions, as shown in Figure 1C. The spatial attention map, $M_{s}\in {\mathbb{R}}^{1\times D\times H\times W}$, is created by concatenating channel-wise average and max pooled features, which are then processed through a convolutional layer:


(12) $$\begin{array}{l} M_{s}=\sigma \left(f_{conv}\left(\text{Concat}\left(\text{GAP}\left(F_{hc}\right),\;\text{GMP}\left(F_{hc}\right)\right)\right)\right), \end{array}$$

where σ is the sigmoid function. The final output feature map is calculated as:


(13) $$\begin{array}{l} F_{out}=M_{s}⊗F_{hc}. \end{array}$$

Compared to the original CBAM, our HBAM offers a structured approach to integrating tabular data into the attention mechanism, enhancing the robustness and semantic relevance of feature refinement in a hybrid-modality context.

#### 3.2.3 Multimodal cascaded attention

**Figure 2:**
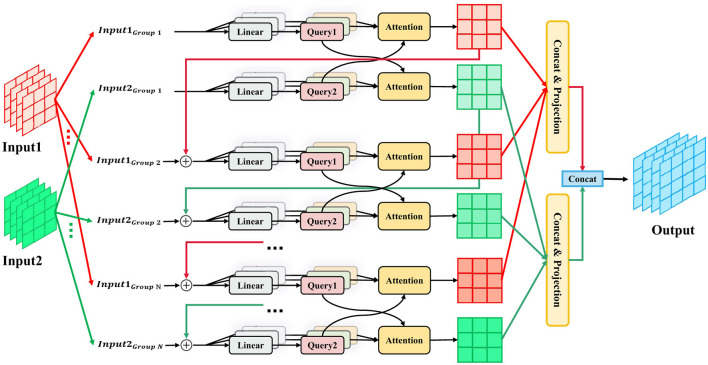
The specific architecture of MultiModal Cascaded Attention. Inputs from two modalities are first divided into *N* groups for independent cross-attention. Then the attention outputs from all groups are concatenated and projected. Two projected features are finally concatenated to form the final representation.

To capture complex, hierarchical dependencies between modalities in multimodal learning, we extend CGA into the multimodal domain with the MMCA module, illustrated in Figure 1D. The feature outputs from the HBAM modules, ***F***_*mri&cli*_ and ***F***_*pet&cli*_, are processed by the MMCA module to produce two modality-enhanced outputs. These are then concatenated and passed through a classification head for the final prediction. Figure 2 provides further insight into the internal workflow and structure of MMCA.

Given two input modalities, ***X*** and ***Y***, each of shape ℝ^*B*×*L*×*C*^, where *B* is the batch size, *L* is the sequence length, and *C* is the number of channels, MMCA first reshapes these inputs to ℝ^*B*×*C*×*L*^ and splits them into *N* groups, corresponding to the number of attention groups. For each group *i* in {1, …, *N*}, a cascaded computation occurs, where the feature representation of the current group builds on features from previous groups, refining attention hierarchically.

For each group, three projections (query, key, value) are derived from the grouped features of both modalities. Let $q_{i}^{x},k_{i}^{x},v_{i}^{x}$ denote the query, key, and value for modality ***X***, and $q_{i}^{y},k_{i}^{y},v_{i}^{y}$ for modality ***Y***. Cross-attention is performed bidirectionally, where features from one modality attend to the keys and values of the other:


(14) $$\begin{array}{l} {\hat{v}}_{i}^{x}=\text{Attention}\left(q_{i}^{y},k_{i}^{x},v_{i}^{x}\right),\;{\hat{v}}_{i}^{y}=\text{Attention}\left(q_{i}^{x},k_{i}^{y},v_{i}^{y}\right). \end{array}$$

The attention operation incorporates a learnable position-aware bias term $b_{i}\in {\mathbb{R}}^{L\times L}$ to enhance spatial sensitivity. The attention score is calculated as:


(15) $$\begin{array}{l} \text{Attention}\left(q_{i},k_{i},v_{i}\right)=\text{Softmax}\left(\frac{q_{i}^{T}k_{i}}{\sqrt{d_{k_{i}}}}+b_{i}\right)v_{i}^{T}, \end{array}$$

where ***d***_*k*_*i*__ is the dimension of the key vectors. Specifically, the Softmax transforms attention logits into a probability distribution across all input positions, which is defined as:


(16) $$\begin{array}{l} \text{Softmax}\left(z_{i}\right)=\frac{\exp\left(z_{i}\right)}{\overset{L}{\underset{j=1}{\sum }}\exp\left(z_{j}\right)}, \end{array}$$

where ***z***_*i*_ denotes the attention logit corresponding to position *i*, and *L* is the total number of positions.

The outputs from all groups are concatenated along the channel dimension and projected to fuse the attended features:


(17) $$\begin{array}{l} Z_{x}={\text{Proj}}_{x}\left(\text{Concat}\left({\hat{v}}_{1}^{x},\ldots ,{\hat{v}}_{H}^{x}\right)\right),\\ Z_{y}={\text{Proj}}_{y}\left(\text{Concat}\left({\hat{v}}_{1}^{y},\ldots ,{\hat{v}}_{H}^{y}\right)\right), \end{array}$$

where $Z_{x},Z_{y}\in {\mathbb{R}}^{B\times L\times C}$ are the fused representations for modalities ***X*** and ***Y***, respectively.

Finally, ***Z***_*x*_ and ***Z***_*y*_ are concatenated along the last dimension to form the final fused representation $Z_{final}\in {\mathbb{R}}^{B\times L\times 2C}$:


(18) $$\begin{array}{l} Z_{final}=\text{Concat}\left(Z_{x},Z_{y}\right). \end{array}$$

The MMCA module offers significant advantages. Its cascaded structure facilitates progressive cross-modal feature fusion, with early attention groups guiding subsequent ones. Moreover, the bidirectional design ensures both modalities are symmetrically enhanced by the other's information, fostering balanced and robust fusion in multimodal contexts.

#### 3.2.4 Loss function

In order to address class imbalance within our dataset, we utilize the focal loss function (Lin et al., 2017). In classification tasks, the cross-entropy (ce) loss is traditionally employed, defined as:


(19) $$\begin{array}{l} L_{ce}=-\left(y\log\left(p\right)+\left(1-y\right)\log\left(1-p\right)\right), \end{array}$$

where *y*∈{0, 1} signifies the ground-truth label, while *p*∈[0, 1] indicates the predicted probability for the positive class. This conventional approach assigns equal weight to both positive and negative samples. Consequently, models often exhibit a bias toward the majority class when faced with class imbalance.

To alleviate this issue, we have adopted the focal loss, which refines the cross-entropy loss by incorporating a dynamic modulating factor. This factor reduces the weight of easily classified samples and concentrates learning on challenging, misclassified instances. The focal loss $L_{focal}$ is expressed as:


(20) $$\begin{array}{l} L_{focal}=-\alpha {\left(1-p\right)}^{\gamma }\left(y\log\left(p\right)+\left(1-y\right)\log\left(1-p\right)\right), \end{array}$$

where α∈[0, 1] serves to balance the significance between positive and negative samples, while γ>0 functions as a focusing parameter, regulating the degree to which easy examples are down-weighted.

## 4 Results

### 4.1 Implementation details

All experiments were conducted using PyTorch version 2.6.0 alongside CUDA 11.8, running on a single NVIDIA V100 32GB GPU. The model underwent training for 200 epochs with a batch size of 8, allowing for efficient data management. For optimizing model parameters, the Adam optimizer was utilized, with the learning rate fixed at 0.0001 to facilitate precise adjustments during training. Given the relatively small size of the datasets, we adopted several strategies to reduce sampling bias and alleviate overfitting. First, we performed five-fold cross-validation to ensure robust evaluation of the model's performance. Secondly, we used a cosine scheduler with a hyperparameter *T*_*max*_ set to 50, allowing for dynamic adjustment of the learning rate throughout the training process and enhancing the model's adaptation capabilities. Additionally, we incorporated an early stopping strategy with a patience value of 50, which effectively prevented overfitting by halting training when the validation loss ceased to improve.

For evaluation, we employ eight metrics to assess both classification performance and model efficiency: Accuracy, Sensitivity, Precision, Area Under the Receiver Operating Characteristic Curve (AUROC), F1-score, Balanced Accuracy, number of parameters (Params), and floating point operations (FLOPs). Given the dataset's class imbalance, we emphasize F1-score and Balanced Accuracy for fairer evaluation. Balanced Accuracy mitigates bias from uneven class distributions and is better suited for imbalanced binary tasks, which is defined as:


(21) $$\begin{array}{l} Balanced\;Accuracy=\frac{1}{2}\left(\frac{\text{TP}}{\text{TP}+\text{FN}}+\frac{\text{TN}}{\text{TN}+\text{FP}}\right), \end{array}$$

where TP, TN, FP, and FN represent the numbers of true positives, true negatives, false positives, and false negatives, respectively.

### 4.2 Comparative experiments

We comprehensively evaluate our framework against representative multimodal approaches under three fusion scenarios. For MRI and PET bimodal fusion, we compare with ResNet+Concat (He et al., 2016), ViT+Concat (Dosovitskiy et al., 2020), nnMamba+Concat (Gong et al., 2025), Diamond (Li et al., 2025), and MDL (Qiu et al., 2024). For MRI and clinical fusion, methods include VAPL (Kang et al., 2023) and HyperFusionNet (Duenias et al., 2025). In the trimodal setting integrating MRI, PET, and clinical data, we compare with IMF (Li et al., 2023), HFBSurv (Li et al., 2022), ITCFN (Hu et al., 2025), and MultimodalADNet (Zhang et al., 2025b). Detailed comparative metrics are presented in Table 2.

**Table 2:** Comparison of various methods on the pMCI vs. sMCI classification task based on five-fold cross-validation.

**Method**	**Modality**			**Performance metrics**							
	**M**	**P**	**C**	**Accuracy (%)** ↑	**Sensitivity (%)** ↑	**Precision (%)** ↑	**AUROC** ↑	**F1-score (%)** ↑	**Balanced accuracy (%)** ↑	**Params (M)** ↓	**FLOPs (G)** ↓
ResNet+Concat (He et al., 2016)	✓	✓		73.75 ± 3.79	75.04±9.16	53.81 ± 5.43	0.7699 ± 0.0276	62.33 ± 4.95	73.26 ± 6.15	66.952	70.924
ViT+Concat (Dosovitskiy et al., 2020)	✓	✓		71.75 ± 3.12	29.09 ± 18.08	42.38 ± 22.15	0.5523 ± 0.0777	33.23 ± 18.82	59.12 ± 6.03	20.853	20.853
nnMamba+Concat (Gong et al., 2025)	✓	✓		73.75 ± 5.04	51.31 ± 16.36	57.81 ± 9.18	0.6978 ± 0.0376	51.75 ± 4.10	66.71 ± 2.17	26.042	48.626
Diamond (Li et al., 2025)	✓	✓		77.76±1.72	48.73 ± 4.09	67.13±6.79	0.7257 ± 0.0304	56.02 ± 1.24	69.19 ± 0.72	23.504	97.638
MDL (Qiu et al., 2024)	✓	✓		71.50 ± 7.39	54.51 ± 17.94	57.29 ± 13.57	0.7130 ± 0.0321	51.65 ± 3.54	66.44 ± 1.98	10.707	19.243
VAPL (Kang et al., 2023)	✓		✓	69.25 ± 4.00	42.25 ± 15.78	48.27 ± 6.03	0.6701 ± 0.0680	42.92 ± 10.81	61.46 ± 5.26	63.504	40.350
HyperFusionNet (Duenias et al., 2025)	✓		✓	75.50 ± 2.69	54.24 ± 8.25	59.96 ± 6.92	0.7330 ± 0.0175	56.05 ± 2.76	69.20 ± 1.50	15.402	47.750
IMF (Li et al., 2023)	✓	✓	✓	77.75 ± 3.98	70.07 ± 9.52	61.81 ± 10.27	0.7946±0.0316	64.34±2.01	75.39±1.41	67.843	70.925
HFBsurv (Li et al., 2022)	✓	✓	✓	75.00 ± 3.26	71.73 ± 5.70	56.12 ± 5.24	0.7552 ± 0.0371	62.54 ± 2.17	74.10 ± 1.37	34.123	141.849
ITCFN (Hu et al., 2025)	✓	✓	✓	75.50 ± 3.76	73.31 ± 3.80	56.40 ± 6.03	0.7750 ± 0.0580	63.58 ± 4.44	74.87 ± 3.23	71.305	71.098
MultimodalADNet (Zhang et al., 2025b)	✓	✓	✓	73.25 ± 4.23	63.93 ± 17.73	57.77 ± 11.18	0.7434 ± 0.0236	57.31 ± 3.53	70.53 ± 2.62	4.320	20.307
**TriLightNet (Ours)**	✓	✓	✓	81.25±0.93	73.91±8.17	65.38±2.91	0.8146±0.0029	69.39±3.76	79.06±2.85	17.405	10.517

The M, P, and C columns indicate the MRI, PET, and Clinical data modalities, respectively. The best and second-best results are in red and blue, respectively.

TriLightNet achieves outstanding performance across several evaluation metrics, with an accuracy of 81.25%, AUROC of 0.8146, and F1-score of 69.39%, all surpassing those of the baseline models. Although ResNet+Concat attains the highest Sensitivity at 75.04%, its relatively low Accuracy and Precision result in suboptimal overall performance. In contrast, TriLightNet ranks second in Sensitivity while maintaining the highest Balanced Accuracy, demonstrating its strong capability to effectively integrate multimodal information and deliver more reliable predictive outcomes.

In terms of computational efficiency, TriLightNet has 17.405 million Params and 10.517 billion FLOPs. While models such as MDL and MultimodalADNet have fewer Params, their predictive performance is significantly inferior. Among the tri-modal approaches, TriLightNet consistently outperforms competing methods, including HFBSurv (34.123 million Params and 141.849 billion FLOPs) and IMF (67.843 million Params and 70.925 billion FLOPs). This efficiency highlights that TriLightNet not only delivers superior accuracy but also exhibits enhanced computational resource efficiency, making it particularly suitable for real-world applications that demand accurate and efficient prediction of cognitive impairment.

### 4.3 Ablation study

We conduct the following two ablation experiments: (1) Assessing the individual contributions of the HBAM and MMCA modules. (2) Evaluating the effect of KAN for the tabular encoder.

The results of ablation experiment (1) are shown in Table 3, demonstrating the significant contributions of both the HBAM and MMCA modules to improve the model's performance. Specifically, HBAM improves the model's accuracy by 0.5%, sensitivity by a remarkable 27.03%, AUROC by 0.1353, and F1-score by 13.91%. These improvements can be attributed to the design of channel attention within the HBAM framework, which facilitates the integration of clinical features into the image feature channels. This interaction at the channel level is followed by spatial attention enhancement, enabling the model to produce more concentrated and informative feature representations.

**Table 3:** Ablation study on the impact of HBAM and MMCA modules.

**Module**		**Performance metrics**					
**HBAM**	**MMCA**	**Accuracy (%)** ↑	**Sensitivity (%)** ↑	**Precision (%)** ↑	**AUROC** ↑	**F1-score (%)** ↑	**Balanced accuracy (%)** ↑
✗	✗	73.50 ± 10.53	48.37 ± 23.95	64.66 ± 16.79	0.6266 ± 0.1591	49.13 ± 17.86	66.01 ± 9.52
✓	✗	74.00 ± 2.67	75.40±7.86	54.60 ± 3.74	0.7619 ± 0.0356	63.04 ± 3.31	74.39 ± 2.89
✗	✓	78.50 ± 4.70	72.94 ± 5.53	63.40 ± 9.76	0.8011 ± 0.0168	67.07 ± 3.99	76.89 ± 2.34
✓	✓	81.25±0.93	73.91 ± 8.17	65.38±2.91	0.8146±0.0029	69.39±3.76	79.06±2.85

The best result is in red.

For MMCA module, it increases accuracy by 5%, AUROC by 0.1745 and balance accuracy by 10.88%. Although there is a slight decrease in precision, the stability of the model improves. This performance can be primarily attributed to two key mechanisms: progressive fusion, which gradually refines dominant features from shallow to deep layers; and bidirectional symmetric enhancement, where each set of cross-attention operations is bidirectional, allowing mutual reinforcement between MRI and PET features, thus achieving comprehensive and balanced multimodal fusion.

Finally, when both HBAM and MMCA modules are jointly applied, the model achieves the best performance across all five evaluation metrics: an accuracy of 81.25%, precision of 65.38%, AUROC of 0.8146, F1-score of 69.39%, and balanced accuracy of 79.06%, demonstrating the complementary benefits and synergistic effect of combining both modules. This indicates that the synergy between these two modules significantly enhances the model's classification capabilities, highlighting the importance of integrating both the HBAM and MMCA modules into the TriLightNet model.

Table 4 presents the ablation results of experiment (2). It illustrates that KAN demonstrates a remarkable advantage over the MLP in clinical tabular feature extraction, achieving consistent improvements across all evaluation metrics. In particular, KAN increases F1-score by 1.72% and balanced accuracy by 1.98%. This improvement can be attributed to KAN's use of expressive B-spline basis functions for modeling clinical features, rather than simple activation functions (e.g., Sigmoid, ReLU) commonly used in MLPs. This enhanced representational capacity allows for better capture of nonlinear patterns in clinical variables, thereby improving overall performance.

**Table 4:** Ablation study on the impact KAN in the hybrid backbone.

**Module**	**Performance metrics**					
	**Accuracy (%)** ↑	**Sensitivity (%)** ↑	**Precision (%)** ↑	**AUROC** ↑	**F1-score (%)** ↑	**Balanced accuracy (%)** ↑
MLP + PoolFormer	80.11 ± 1.55	70.84 ± 5.50	65.22 ± 4.87	0.8101 ± 0.0118	67.67 ± 2.62	77.08 ± 2.03
KAN + PoolFormer	81.25±0.93	73.91±8.17	65.38±2.91	0.8146±0.0029	69.39±3.76	79.06±2.85

The best result is in red.

### 4.4 Model visualization and interpretability

#### 4.4.1 Metrics visualization

**Figure 3:**
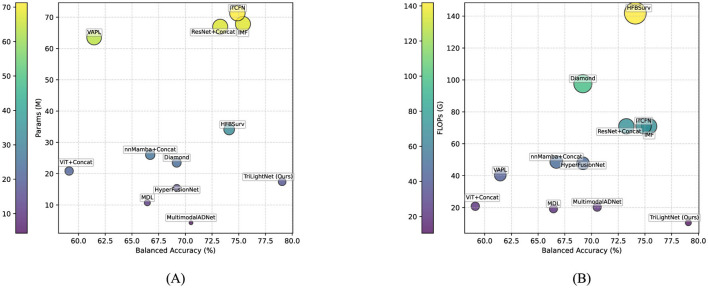
Model metrics bubble chart. **(A)** Balanced accuracy vs. Params. **(B)** Balanced accuracy vs. FLOPs.

To clearly illustrate the effectiveness of our model, we present a visual comparison of key evaluation metrics using bubble charts, as depicted in Figure 3. We focused on three crucial metrics: Balanced Accuracy, Params, and FLOPs.

Figure 3A shows the relationship between Balanced Accuracy and Params. TriLightNet stands out by achieving excellent performance with a relatively low parameter count, underscoring its efficiency. Figure 3B compares Balanced Accuracy with FLOPs, further showcasing TriLightNet's lightweight design and high performance.

**Figure 4:**
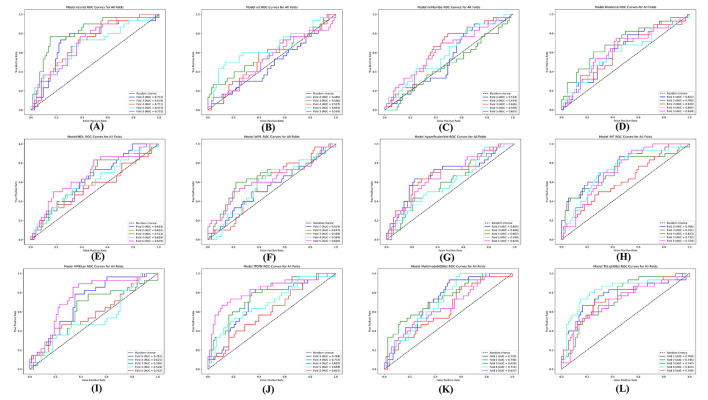
Test set performance for each of the five models trained during five-fold cross-validation. **(A)** Resnet + Concat. **(B)** ViT + Concat. **(C)** nnMamba + Concat. **(D)** Diamond. **(E)** MDL. **(F)** VAPL. **(G)** hyperfusionNet. **(H)** IMF. **(I)** HFBSurv. **(J)** ITCFN. **(K)** MultimodalADNet. **(L)** TriLightNet (Ours).

Additionally, we assessed the robustness and generalization capabilities of each model by testing the trained models from each fold of the five-fold cross-validation on a test set. The Receiver Operating Characteristic (ROC) curves for each fold are presented in Figure 4.

From Figure 4, we observe that certain triple-modal approaches, such as IMF and ITCFN, show significant fluctuations in their AUC scores across different folds, suggesting potential instability. In contrast, our proposed method, TriLightNet, displays consistently high AUC results across all five folds. This consistency provides strong empirical evidence of TriLightNet's superior generalization capability and robustness when handling diverse validation subsets.

#### 4.4.2 Model interpretability

To improve the interpretability of our model and gain insight into how various input modalities affect the final decision, we utilize the IG method (Sundararajan et al., 2017), a common technique for interpreting deep neural networks. We specifically apply IG to MRI and PET image modalities to produce attribution maps that display the contribution of each voxel to the model's predictions. These maps are then overlaid on the original MRI and PET images for visualization. For a fair comparison, we also apply the same IG-based interpretability method to several representative multimodal fusion baselines, including IMF, ITCFN, MultimodalADNet, and HFBSurv. This allows us to compare the spatial attention patterns across models under identical conditions.

**Figure 5:**
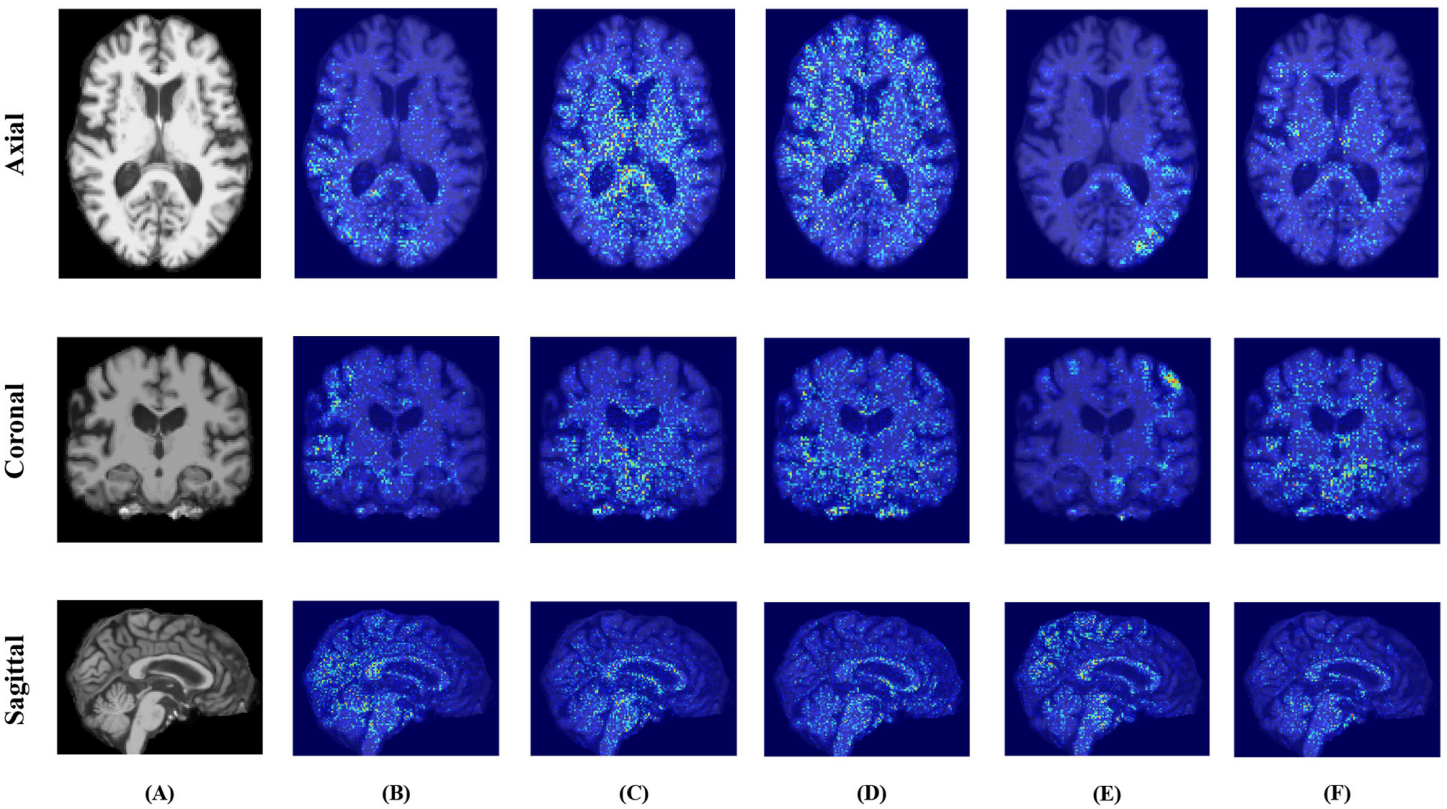
Comparison of Integrated Gradients attribution maps on MRI across different models. **(A)** Original. **(B)** IMF. **(C)** ITCFN. **(D)** MultimodalADNet. **(E)** HFBSurv. **(F)** TriLightNet (Ours).

**Figure 6:**
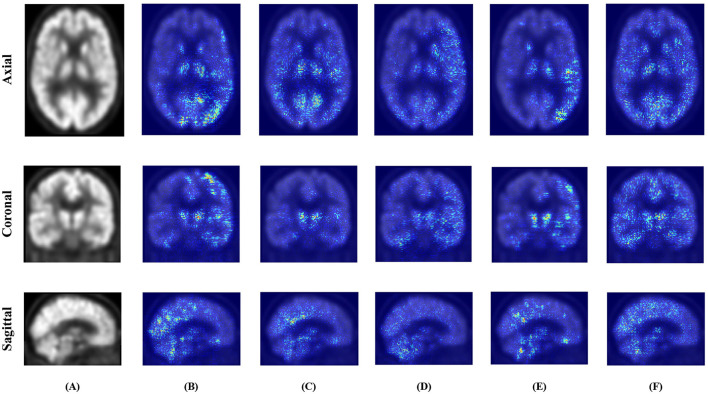
Comparison of Integrated Gradients attribution maps on PET across different models. **(A)** Original. **(B)** IMF. **(C)** ITCFN. **(D)** MultimodalADNet. **(E)** HFBSurv. **(F)** TriLightNet (Ours).

As illustrated in Figures 5, 6, the attribution maps reveal distinct attention patterns across different models. For instance, TriLightNet (Ours) tends to focus on clinically relevant regions such as the hippocampus and posterior cingulate cortex, which are known to be associated with Alzheimer's disease progression. In contrast, some baseline models exhibit more diffused or inconsistent attention patterns. These findings suggest that our model not only achieves superior performance but also offers more focused and biologically plausible interpretability.

## 5 Discussion

The TriLightNet, a novel lightweight triple-modal fusion network, demonstrated superior performance in predicting MCI-to-AD progression. Its strong performance can be attributed to synergistic modules: the HBAM for nuanced cross-modal feature interaction and the MMCA for efficient, hierarchical data integration, both validated by ablation studies. Compared to competing methods, TriLightNet not only surpassed existing methods in accuracy and AUROC but also achieved this with significantly lower computational costs (Params and FLOPs), highlighting its practical value in real-world clinical settings where computational resources are often limited.

Furthermore, the IG provided valuable insights into the decision-making process of TriLightNet. The attribution maps demonstrated that TriLightNet consistently focused on clinically relevant brain regions across both MRI and PET modalities, including the hippocampus, medial temporal lobe, and posterior cingulate cortex, which are affected early in AD. Notably, while competing models such as IMF and ITCFN showed reasonable focus on disease-related regions in MRI, they exhibited weaker and more diffuse attention patterns in PET images. In contrast, TriLightNet maintained clear and concentrated attribution in both MRI and PET, indicating its superior ability to extract complementary structural and functional information. It suggests that TriLightNet achieves not only better predictive accuracy but also more biologically plausible feature learning, potentially enhancing its interpretability and clinical trustworthiness.

Despite these promising results, several limitations should be acknowledged. First, although we leveraged the ADNI dataset for evaluation, external validation on independent cohorts is necessary to assess generalizability across diverse populations and imaging protocols. Second, TriLightNet currently requires complete multimodal data, future work will focus on extending the framework to handle incomplete modality scenarios, which are common in real-world clinical practice. Third, while KAN was utilized as an efficient feature extractor for tabular data, its intrinsic interpretability was not fully explored. Future work may further investigate KAN's potential to reveal nonlinear relationships between clinical variables and AD progression.

## 6 Conclusion

We introduced TriLightNet, a novel and efficient triple-modal fusion network for predicting cognitive decline in AD by integrating MRI, PET, and clinical tabular data. Extensive experiments on the ADNI dataset demonstrated TriLightNet's superior classification performance over state-of-the-art multimodal methods, alongside significant reductions in parameter count and computational cost. Key contributions include a hybrid KAN-PoolFormer backbone for efficient tabular feature extraction, an HBAM for enhanced imaging-clinical data interactions, and an MMCA for progressive cross-modal fusion. Beyond state-of-the-art results, TriLightNet offers interpretability via IG-based attribution maps, highlighting disease-relevant brain regions and underscoring its potential for aiding timely clinical interventions in AD progression. Future work will focus on adapting TriLightNet to handle incomplete or missing modalities and validating its generalizability across multi-center datasets. Additionally, we plan to explore the standalone application of the KAN network to clinical datasets for predictive modeling and interpretability analysis through visualization.

## Data Availability

The data used in this study are publicly available from the Alzheimer's Disease Neuroimaging Initiative (ADNI) database: http://adni.loni.usc.edu/. Researchers can apply for access to the ADNI data by registering and submitting a data access request through the ADNI Data Sharing and Publications Committee. All data used in this work, including structural MRI, PET scans, and clinical information, were obtained following ADNI's data usage policies and guidelines.

## References

[B1] Atitallah S. B. Driss M. Boulila W. Koubaa A. (2024). Enhancing early Alzheimer's disease detection through big data and ensemble few-shot learning. IEEE J. Biomed. Health Inf . 1–12. 10.1109/JBHI.2024.3473541 39356607

[B2] Baytaş İ. M. (2024). Predicting progression from mild cognitive impairment to Alzheimer's dementia with adversarial attacks. IEEE J. Biomed. Health Inf. 28, 3750–3761. 10.1109/JBHI.2024.3373703 38507374 PMC12476489

[B3] Dao D.-P. Yang H.-J. Kim J. Ho N.-H. the Alzheimer's Disease Neuroimaging Initiative (2025). Longitudinal Alzheimer's disease progression prediction with modality uncertainty and optimization of information flow. IEEE J. Biomed. Health Inf. 29, 259–272. 10.1109/JBHI.2024.3472462 39356605 PMC11875883

[B4] Ding T. Xiang D. Schubert K. E. Dong L. (2025). Gkan: Explainable diagnosis of Alzheimer's disease using graph neural network with Kolmogorov-Arnold networks. arXiv [Preprint]. arXiv:2504.00946. 10.48550/arXiv.2504.00946

[B5] Dosovitskiy A. Beyer L. Kolesnikov A. Weissenborn D. Zhai X. Unterthiner T.. (2020). An image is worth 16x16 words: transformers for image recognition at scale. arXiv [Preprint]. arXiv:2010.11929. 10.48550/arXiv.2010.11929

[B6] Duenias D. Nichyporuk B. Arbel T. Raviv T. R. (2025). Hyperfusion: a hypernetwork approach to multimodal integration of tabular and medical imaging data for predictive modeling. Med. Image Anal. 102:103503. 10.1016/j.media.2025.103503 40037055

[B7] Elazab A. Wang C. Abdelaziz M. Zhang J. Gu J. Górriz J. M.. (2024). Alzheimer's disease diagnosis from single and multimodal data using machine and deep learning models: achievements and future directions. Expert Syst. Appl. 255:124780. 10.1016/j.eswa.2024.124780

[B8] El-Gamal F. E. A. Elmogy M. Mahmoud A. Shalaby A. Switala A. E. Ghazal M.. (2021). A personalized computer-aided diagnosis system for mild cognitive impairment (MCI) using structural MRI (sMRI). Sensors 21:5416. 10.3390/s21165416 34450858 PMC8400990

[B9] Eslamian A. Aghaei A. A. Cheng Q. (2025). Tabkan: advancing tabular data analysis using Kolmogorov-Arnold network. arXiv [Preprint]. arXiv:2504.06559. 10.48550/arXiv.2504.06559

[B10] Francesconi A. di Biase L. Cappetta D. Rebecchi F. Soda P. Sicilia R.. (2025). Class balancing diversity multimodal ensemble for Alzheimer's disease diagnosis and early detection. Comput. Med. Imaging Graph. 123:102529. 10.1016/j.compmedimag.2025.102529 40147216

[B11] Gao W. Gong Z. Deng Z. Rong F. Chen C. Ma L.. (2024). Tabkanet: tabular data modeling with Kolmogorov-Arnold network and transformer. arXiv [Preprint]. arXiv:2409.08806. 10.48550/arXiv.2409.08806

[B12] Gaser C. Franke K. Klöppel S. Koutsouleris N. Sauer H. Initiative A. D. N.. (2013). Brainage in mild cognitive impaired patients: predicting the conversion to Alzheimer's disease. PLoS ONE 8:e0067346. 10.1371/journal.pone.0067346 23826273 PMC3695013

[B13] Gong H. Kang L. Wang Y. Wang Y. Wan X. Wu X.. (2025). “NNMAMBA: 3D biomedical image segmentation, classification and landmark detection with state space model,” in 2025 IEEE 22nd International Symposium on Biomedical Imaging (ISBI) (Houston, TX: IEEE), 1–5. 10.1109/ISBI60581.2025.10980694

[B14] Grigas O. Maskeliunas R. Damaševičius R. (2024). Early detection of dementia using artificial intelligence and multimodal features with a focus on neuroimaging: a systematic literature review. Health Technol. 14, 201–237. 10.1007/s12553-024-00823-040286904

[B15] Gryshchuk V. Singh D. Teipel S. Dyrba M. (2025). Contrastive self-supervised learning for neurodegenerative disorder classification. Front. Neuroinform. 19:1527582. 10.3389/fninf.2025.1527582 40034453 PMC11873101

[B16] Haq E. U. Yong Q. Yuan Z. Huarong X. Haq R. U. (2025). Multimodal fusion diagnosis of the Alzheimer's disease via lightweight CNN-LSTM model using magnetic resonance imaging (MRI). Biomed. Signal Process. Control 104:107545. 10.1016/j.bspc.2025.107545

[B17] He K. Zhang X. Ren S. Sun J. (2016). “Deep residual learning for image recognition,” in Proceedings of the IEEE Conference on Computer Vision and Pattern Recognition (CVPR) (Las Vegas, NV: IEEE), 770–778. 10.1109/CVPR.2016.90

[B18] Hu J. Shen L. Sun G. (2018). “Squeeze-and-excitation networks,” in Proceedings of the IEEE Conference on Computer Vision and Pattern Recognition (CVPR) (Salt Lake City, UT: IEEE), 7132–7141. 10.1109/CVPR.2018.00745

[B19] Hu X. Shen X. Sun Y. Shan X. Min W. Su L.. (2025). “ITCFN: incomplete triple-modal co-attention fusion network for mild cognitive impairment conversion prediction,” in 2025 IEEE 22nd International Symposium on Biomedical Imaging (ISBI) (Houston, TX: IEEE), 1–5. 10.1109/ISBI60581.2025.10980706

[B20] Jabason E. Ahmad M. O. Swamy M. (2025). A lightweight deep convolutional neural network extracting local and global contextual features for the classification of Alzheimer's disease using structural MRI. IEEE J. Biomed. Health Inf. 29, 2061–2073. 10.1109/JBHI.2024.3512417 40030424

[B21] Kang L. Gong H. Wan X. Li H. (2023). “Visual-attribute prompt learning for progressive mild cognitive impairment prediction,” in International Conference on Medical Image Computing and Computer-Assisted Intervention (Cham: Springer), 547–557. 10.1007/978-3-031-43904-9_53

[B22] Kohlbrenner M. Bauer A. Nakajima S. Binder A. Samek W. Lapuschkin S.. (2020). “Towards best practice in explaining neural network decisions with LRP,” in 2020 International Joint Conference on Neural Networks (IJCNN) (Glasgow: IEEE), 1–7. 10.1109/IJCNN48605.2020.9206975

[B23] Lei Z. Zhu W. Liu J. Hua C. Li J. Shah S. A. A.. (2024). RLAD: a reliable hippo-guided multi-task model for Alzheimer's disease diagnosis. IEEE J. Biomed. Health Inf . 1–12. 10.1109/JBHI.2024.3412926 38861438

[B24] Li R. Wu X. Li A. Wang M. (2022). HFBSURV: hierarchical multimodal fusion with factorized bilinear models for cancer survival prediction. Bioinformatics 38, 2587–2594. 10.1093/bioinformatics/btac113 35188177 PMC9048674

[B25] Li X. Zhao X. Xu J. Zhang Y. Xing C. (2023). “IMF: interactive multimodal fusion model for link prediction,” in Proceedings of the ACM Web Conference 2023 (New York, NY: ACM), 2572–2580. 10.1145/3543507.3583554

[B26] Li Y. Ghahremani M. Wally Y. Wachinger C. (2025). “Diamond: dementia diagnosis with multi-modal vision transformers using MRI and pet,” in 2025 IEEE/CVF Winter Conference on Applications of Computer Vision (WACV) (Tucson, AZ: IEEE), 107–116. 10.1109/WACV61041.2025.00021

[B27] Lin T.-Y. Goyal P. Girshick R. He K. Dollár P. (2017). “Focal loss for dense object detection,” in Proceedings of the IEEE International Conference on Computer Vision (Venice: IEEE), 2980–2988. 10.1109/ICCV.2017.324

[B28] Liu X. Peng H. Zheng N. Yang Y. Hu H. Yuan Y.. (2023). “Efficientvit: memory efficient vision transformer with cascaded group attention,” in Proceedings of the IEEE Conference on Computer Vision and Pattern Recognition (CVPR) (Vancouver, BC: IEEE), 14420–14430. 10.1109/CVPR52729.2023.01386

[B29] Liu Y. Yue L. Xiao S. Yang W. Shen D. Liu M.. (2022). Assessing clinical progression from subjective cognitive decline to mild cognitive impairment with incomplete multi-modal neuroimages. Med. Image Anal. 75:102266. 10.1016/j.media.2021.102266 34700245 PMC8678365

[B30] Liu Z. Wang Y. Vaidya S. Ruehle F. Halverson J. Soljačić M.. (2024). Kan: Kolmogorov-Arnold networks. arXiv [Preprint]. arXiv:2404.19756. 10.48550/arXiv.2404.19756

[B31] Mathew J. Mekkayil L. Ramasangu H. Karthikeyan B. R. Manjunath A. G. (2016). “Robust algorithm for early detection of Alzheimer's disease using multiple feature extractions,” in 2016 IEEE Annual India Conference (INDICON) (Bangalore: IEEE), 1–6. 10.1109/INDICON.2016.7839026

[B32] Moradi E. Pepe A. Gaser C. Huttunen H. Tohka J. (2015). Machine learning framework for early mri-based Alzheimer's conversion prediction in MCI subjects. Neuroimage 104, 398–412. 10.1016/j.neuroimage.2014.10.002 25312773 PMC5957071

[B33] Mueller S. G. Weiner M. W. Thal L. J. Petersen R. C. Jack C. R. Jagust W.. (2005). Ways toward an early diagnosis in Alzheimer's disease: the Alzheimer's disease neuroimaging initiative (ADNI). Alzheimers Dement. 1, 55–66. 10.1016/j.jalz.2005.06.003 17476317 PMC1864941

[B34] Peng L. Cai S. Wu Z. Shang H. Zhu X. Li X.. (2024). MMGPL: multimodal medical data analysis with graph prompt learning. Med. Image Anal. 97:103225. 10.1016/j.media.2024.103225 38908306

[B35] Poeta E. Giobergia F. Pastor E. Cerquitelli T. Baralis E. (2024). “A benchmarking study of Kolmogorov-Arnold networks on tabular data,” in In 2024 IEEE 18th International Conference on Application of Information and Communication Technologies (AICT) (Turin: IEEE), 1–6. 10.1109/AICT61888.2024.10740444

[B36] Qiu Z. Yang P. Xiao C. Wang S. Xiao X. Qin J.. (2024). 3D multimodal fusion network with disease-induced joint learning for early Alzheimer's disease diagnosis. IEEE Trans. Med. Imaging 43, 3161–3175. 10.1109/TMI.2024.3386937 38607706

[B37] Selvaraju R. R. Cogswell M. Das A. Vedantam R. Parikh D. Batra D.. (2017). “Grad-cam: visual explanations from deep networks via gradient-based localization,” in Proceedings of the IEEE International Conference on Computer Vision (Venice: IEEE), 618–626. 10.1109/ICCV.2017.74

[B38] Sundararajan M. Taly A. Yan Q. (2017). “Axiomatic attribution for deep networks,” in International Conference on Machine Learning, Volume 70 (PMLR), 3319–3328.

[B39] Tan M. Le Q. V. (2019). “Efficientnet: rethinking model scaling for convolutional neural networks,” in Proceedings of the 36th International Conference on Machine Learning, ICML 2019, 9-15 June 2019, Long Beach, California, USA, Volume 97 of Proceedings of Machine Learning Research, eds. K. Chaudhuri, and R. Salakhutdinov (Long Beach, CA: PMLR), 6105–6114.35077359

[B40] Verma A. Verma A. Sharma R. P. (2025). VGG-KAN: A Hybrid Approach for Alzheimer's Disease Diagnosis using Kolmogorov Arnold Network. 10.36227/techrxiv.174803834.41610666/v1

[B41] Wang C. Lei Y. Chen T. Zhang J. Li Y. Shan H.. (2024). Hope: hybrid-granularity ordinal prototype learning for progression prediction of mild cognitive impairment. IEEE J. Biomed. Health Inf. 28, 6429–6440. 10.1109/JBHI.2024.3357453 38261490

[B42] Wang D. Honnorat N. Fox P. T. Ritter K. Eickhoff S. B. Seshadri S.. (2023). Deep neural network heatmaps capture Alzheimer's disease patterns reported in a large meta-analysis of neuroimaging studies. Neuroimage 269:119929. 10.1016/j.neuroimage.2023.119929 36740029 PMC11155416

[B43] Wang Y. (2025). “Application of Kolmogorov-Arnold networks combined with graph convolutional networks in Alzheimer's disease diagnosis,” in 2025 5th International Conference on Consumer Electronics and Computer Engineering (ICCECE) (Dongguan: IEEE), 204–207. 10.1109/ICCECE65250.2025.10985338

[B44] Wen J. Thibeau-Sutre E. Diaz-Melo M. Samper-González J. Routier A. Bottani S.. (2020). Convolutional neural networks for classification of Alzheimer's disease: overview and reproducible evaluation. Med. Image Anal. 63:101694. 10.1016/j.media.2020.101694 32417716

[B45] Woo S. Park J. Lee J.-Y. Kweon I. S. (2018). “CBAM: convolutional block attention module,” in Proceedings of the European Conference on Computer Vision (ECCV), Volume 11211 (Cham: Springer), 3–19. 10.1007/978-3-030-01234-2_1

[B46] Yu W. Luo M. Zhou P. Si C. Zhou Y. Wang X.. (2022). “Metaformer is actually what you need for vision,” in Proceedings of the IEEE Conference on Computer Vision and Pattern Recognition (CVPR) (New Orleans, LA: IEEE), 10819–10829. 10.1109/CVPR52688.2022.01055

[B47] Yun S. Choi I. Peng J. Wu Y. Bao J. Zhang Q.. (2024). Flex-MOE: modeling arbitrary modality combination via the flexible mixture-of-experts. arXiv [Preprint]. arXiv:2410.08245. 10.48550/arXiv.2410.08245

[B48] Zhang J. Wu X. Tang X. Zhou L. Wang L. Wu W.. (2025a). Asynchronous functional brain network construction with spatiotemporal transformer for MCI classification. IEEE Trans. Med. Imaging 44, 1168–1180. 10.1109/TMI.2024.3486086 39446548

[B49] Zhang Y. Sun K. Liu Y. Xie F. Guo Q. Shen D.. (2025b). A modality-flexible framework for Alzheimer's disease diagnosis following clinical routine. IEEE J. Biomed. Health Inf. 29, 535–546. 10.1109/JBHI.2024.3472011 39352829

